# Supplementary material for: The PYRIN domain-only protein POP2 inhibits inflammasome priming and activation
Source: Nat Commun. 2017 Jun 5;8:15556. doi: 10.1038/ncomms15556 (PMC5465353; doi:10.1038/ncomms15556)
Supplement: Supplementary Information — Supplementary Figures and Supplementary Table 1 [file ncomms15556-s1.pdf]

**Supplementary Figures**

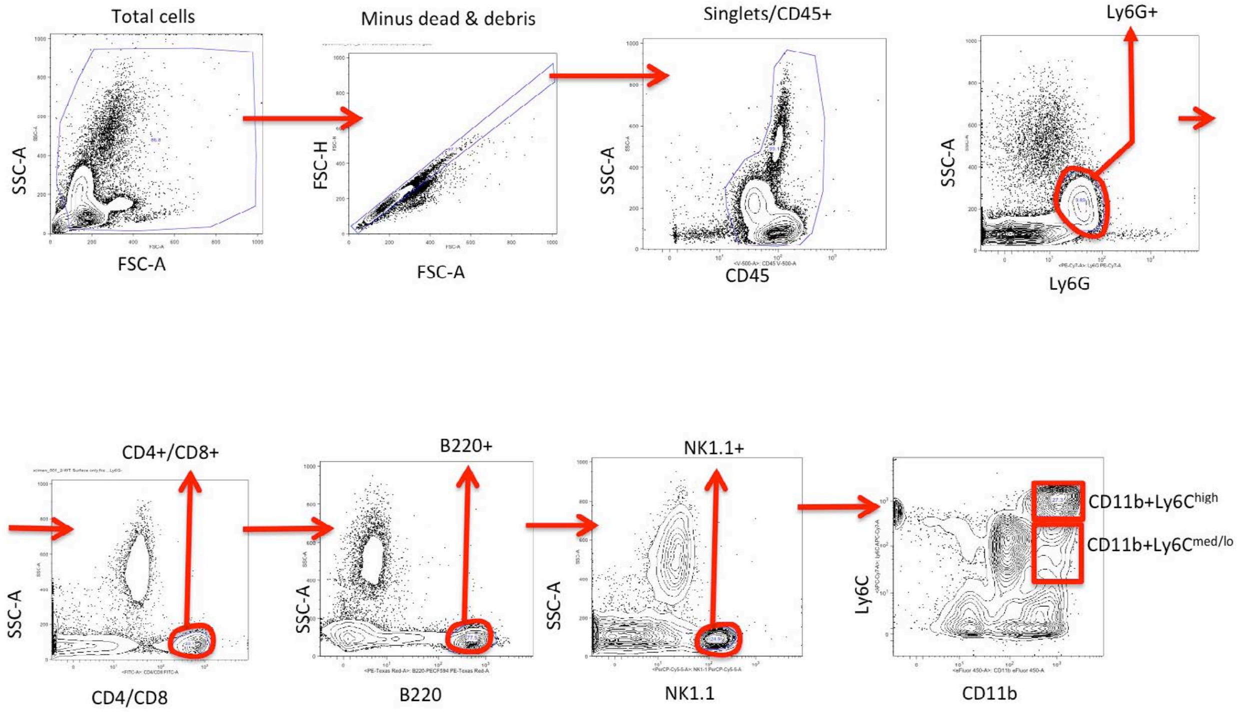


**Supplementary Figure 1 | FACS gating strategy** FACS gating for the characterization of POP2 expression using Cy5-labeled SmartFlares in peripheral blood cells, which corresponds to Figure 1b.

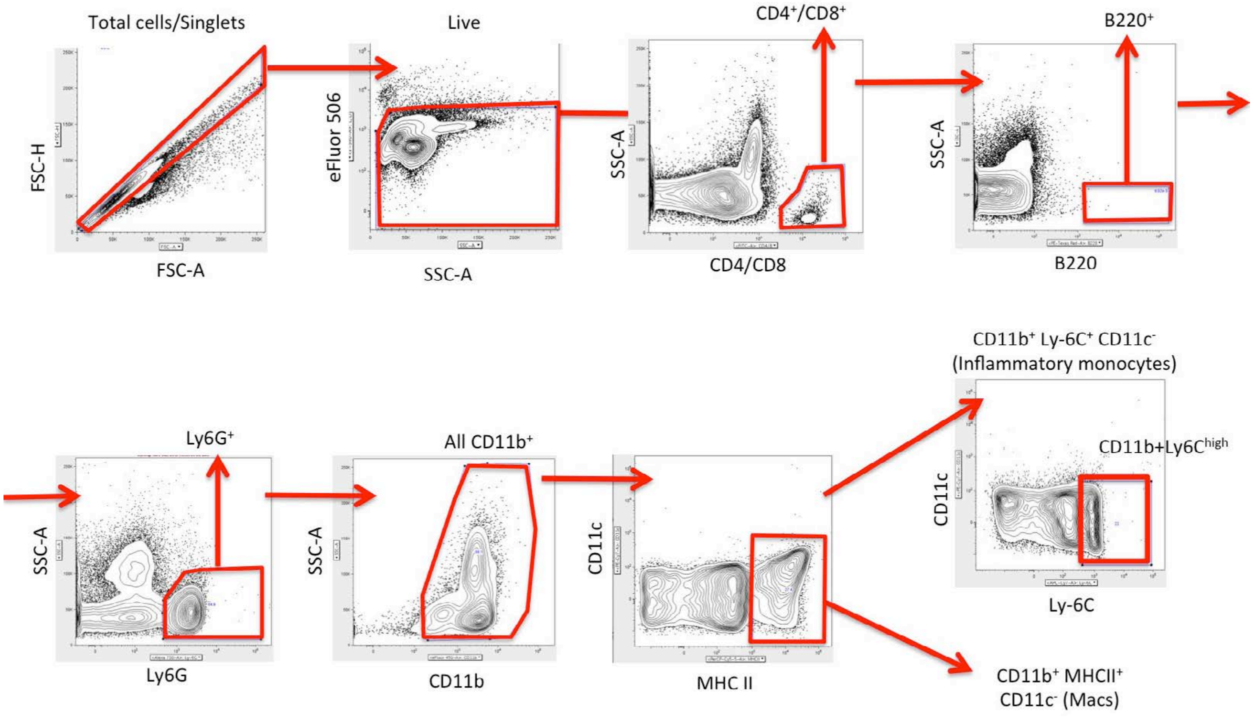


**Supplementary Figure 2 | FACS gating strategy** FACS gating strategy for the phenotypic characterization of airpouch exudate cells, which corresponds to Figure 1h.

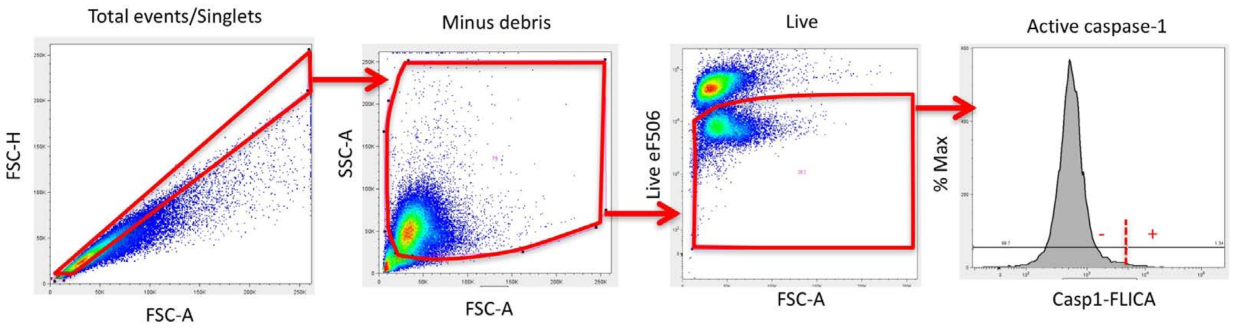


**Supplementary Figure 3 | FACS gating strategy** FACS gating strategy for the Caspase-1 FLICA assay in BMDM, which corresponds to Figure 2c, d.

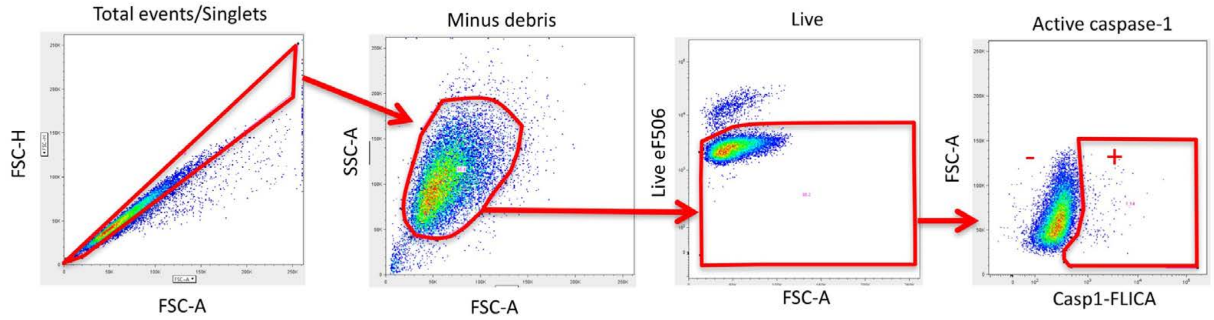


**Supplementary Figure 4 | FACS gating strategy** FACS gating strategy for the Caspase-1 FLICA assay in THP-1 cells, which corresponds to Figure 4a, b, e.

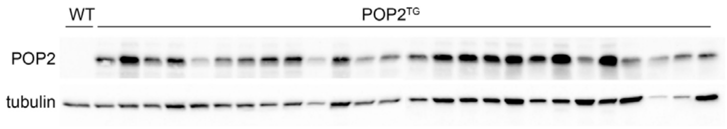


**Supplementary Figure 5 | Quantification of POP2 protein expression** Quantification of POP2 protein expression by immunoblot in macrophages differentiated ex vivo from peripheral blood monocytes, showing a representative result, which corresponds to Fig. 6g.

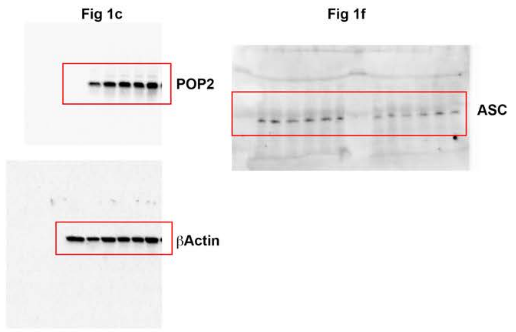


**Supplementary Figure 6 | Uncropped western blots for Figure 1 are shown.**

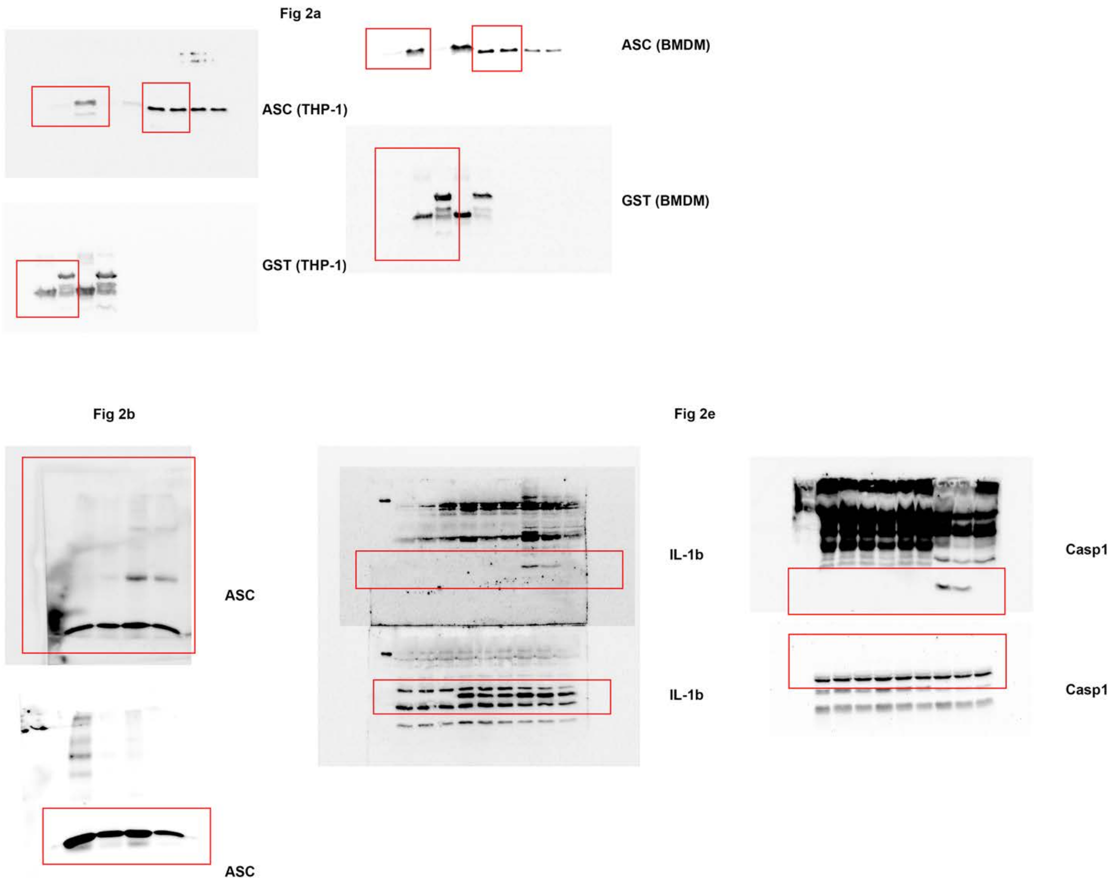


**Supplementary Figure 7 | Uncropped western blots for Figure 2 are shown.**

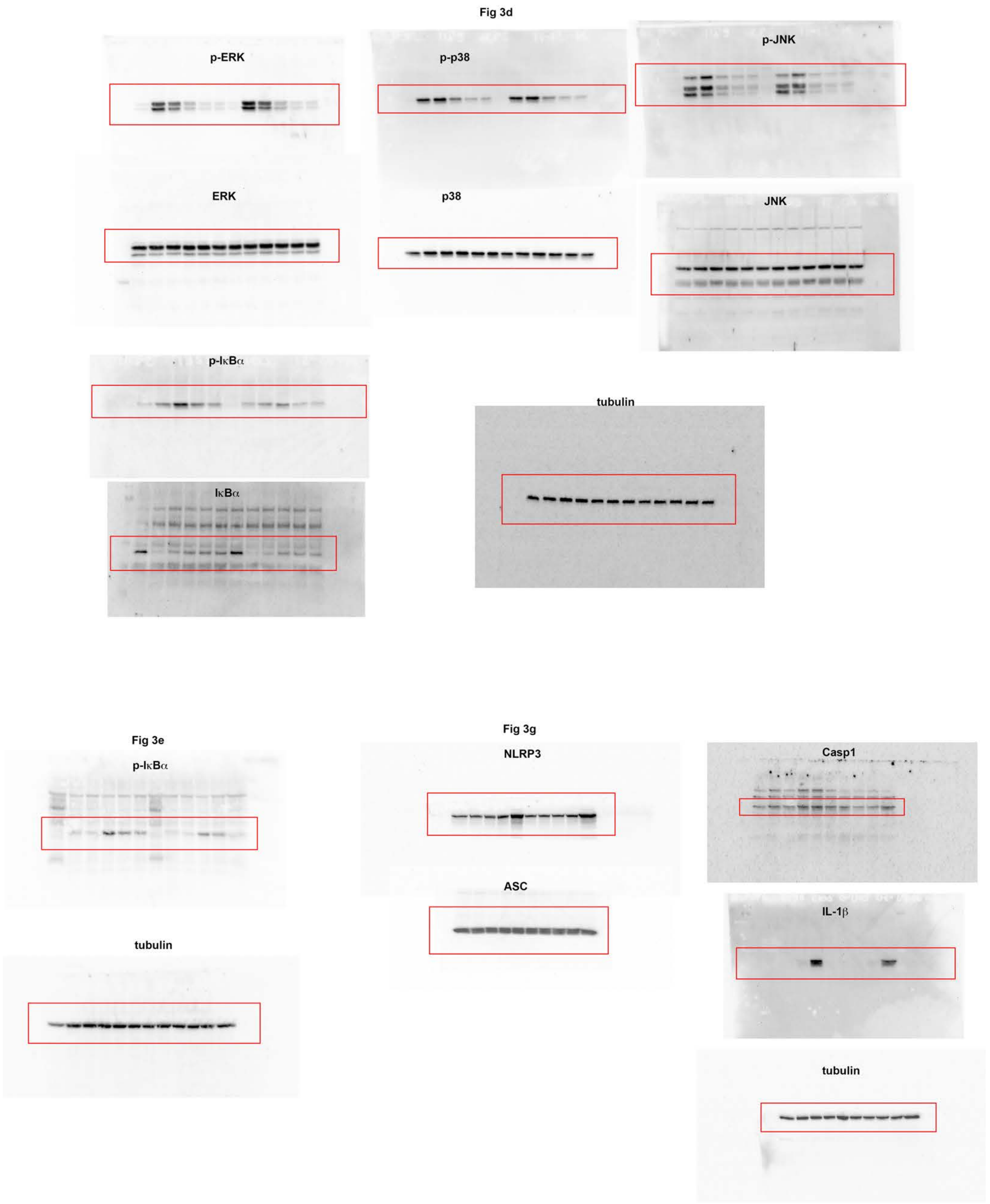


**Supplementary Figure 8 | Uncropped western blots for Figure 3 are shown.**

Fig 4l

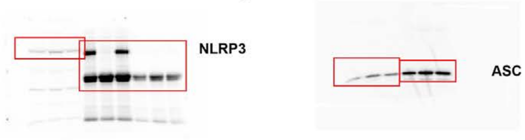


**Supplementary Figure 9 | Uncropped western blots for Figure 4 are shown.**

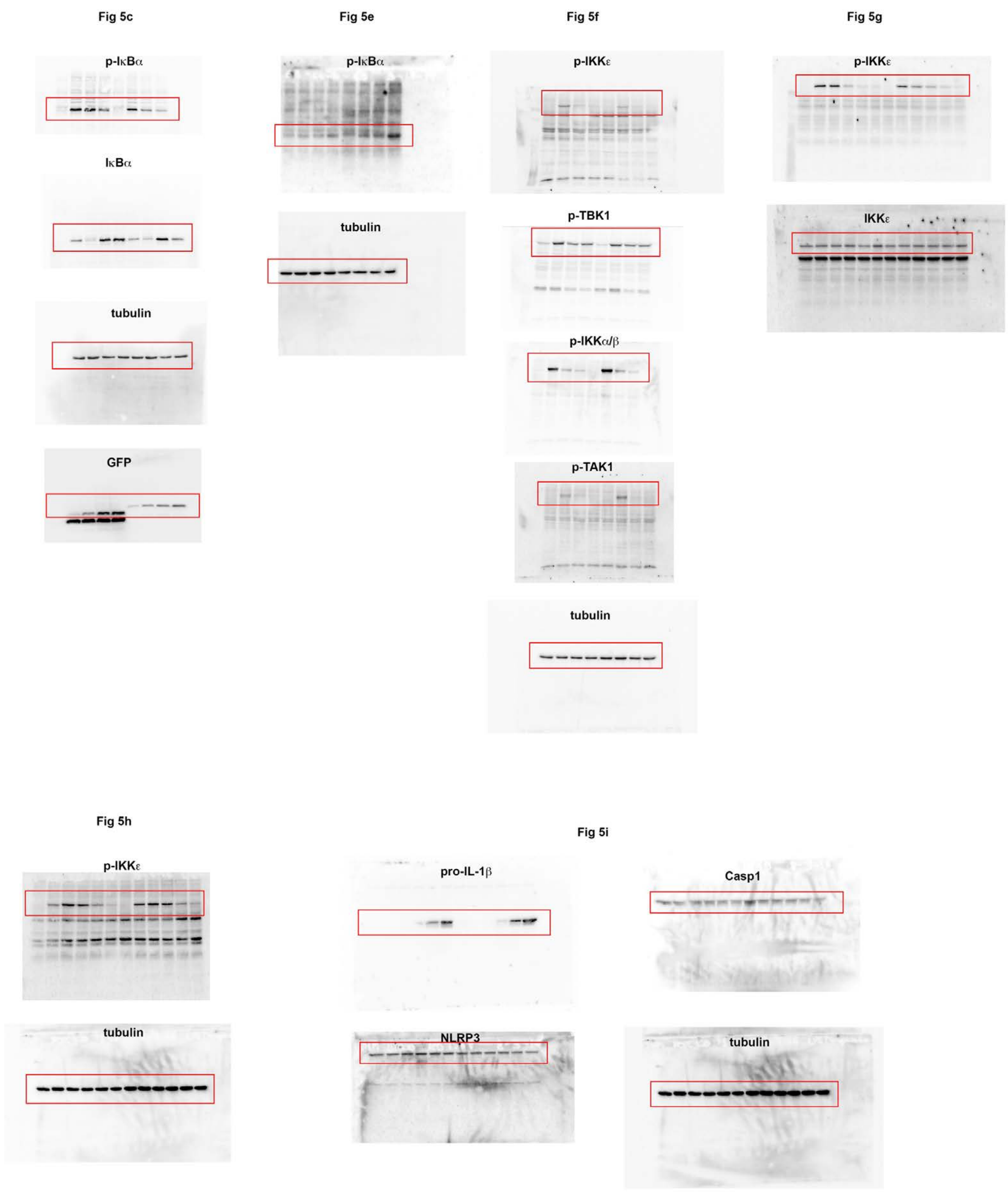


**Supplementary Figure 10 | Uncropped western blots for Figure 5 are shown.**
